# Small Bowel Obstruction Caused by an Aggressive Weight Loss Diet in a Patient With No Predisposing Factors

**DOI:** 10.7759/cureus.32594

**Published:** 2022-12-16

**Authors:** Samara C Hassranah, Aryaa Ramnarine, Sangeeta Parbhu, Vijay Naraynsingh

**Affiliations:** 1 Department of Surgery, Medical Associates Hospital, St. Joseph, TTO; 2 Clinical Surgical Sciences, UWI (The University of the West Indies), St. Augustine, TTO; 3 Department of Surgery, Sangre Grande Hospital, Sangre Grande, TTO

**Keywords:** enterotomy, ileotomy, small-bowel obstruction, healthy foods, plant-based diet, high fibre diet, phytobezoar, bezoar, gastrointestinal obstruction, small intestinal obstruction

## Abstract

Phytobezoars are a well-documented cause of small bowel obstruction. Previous reports include patients who have predisposing factors such as gastric surgery, diabetes mellitus, or poor dentition. Consequences of extreme dieting have also been reported, but a resultant phytobezoar and life-threatening bowel obstruction are rare. We present a case of phytobezoar solely due to a diet inordinately high in fiber.

## Introduction

Phytobezoars are well known to occur in patients with poor dentition, previous gastric surgery, and diabetes mellitus [1]. However, there are only a few reports of them occurring due to diet changes aimed at achieving weight loss. In recent history, the number of people on extreme or ‘fad’ diets has increased, but the consequence of a phytobezoar secondary to a weight loss diet has yet to be fully documented. Doctors need to be aware of this rare complication. We present a case of a patient who went on an extreme diet for weight loss and the resultant intestinal obstruction.

## Case presentation

A 48-year-old male presented with two days of worsening, colicky abdominal pain, bloating, and marked anorexia. The bloating worsened, and he vomited twice on the day of admission. He was stable with a benign abdomen, except for mild distension, and had normal vital signs. He passed no stool in the preceding 36 hours. He had no previous surgeries.

One year prior, he was diagnosed with hypertension and kidney failure and went on a weight loss diet in an attempt to achieve a healthier lifestyle. He added high volumes of vegetables and fruits to his diet, two dinner platefuls a day of raw broccoli, carrots, cauliflower, celery stalks, cucumber, sweet pepper, watercress, mangoes, and watermelon. He also ate cooked, high-fiber foods, dasheen, yam, corn, and green bananas. He lost 50 pounds in 12 months with his BMI decreasing from 35.8 kg/m^2 ^to 28.7 kg/m^2^.

**Figure 1:**
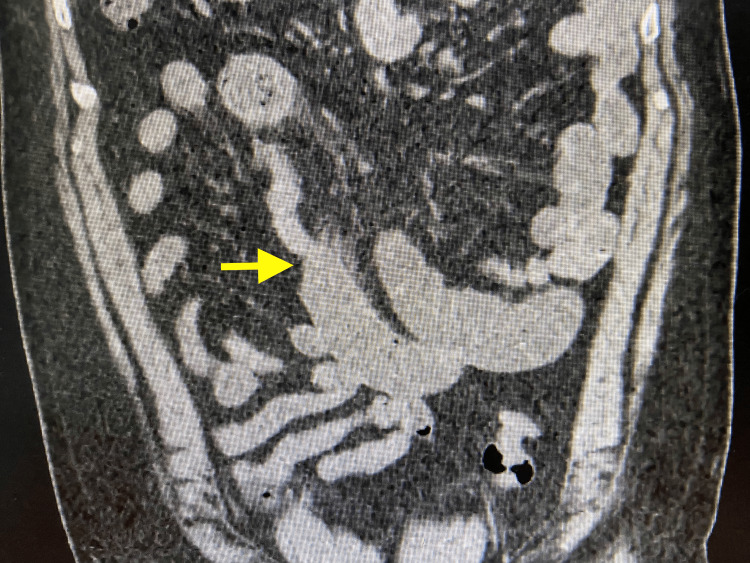
Transition point in the distal ileum on CT scan (arrow)

**Figure 2:**
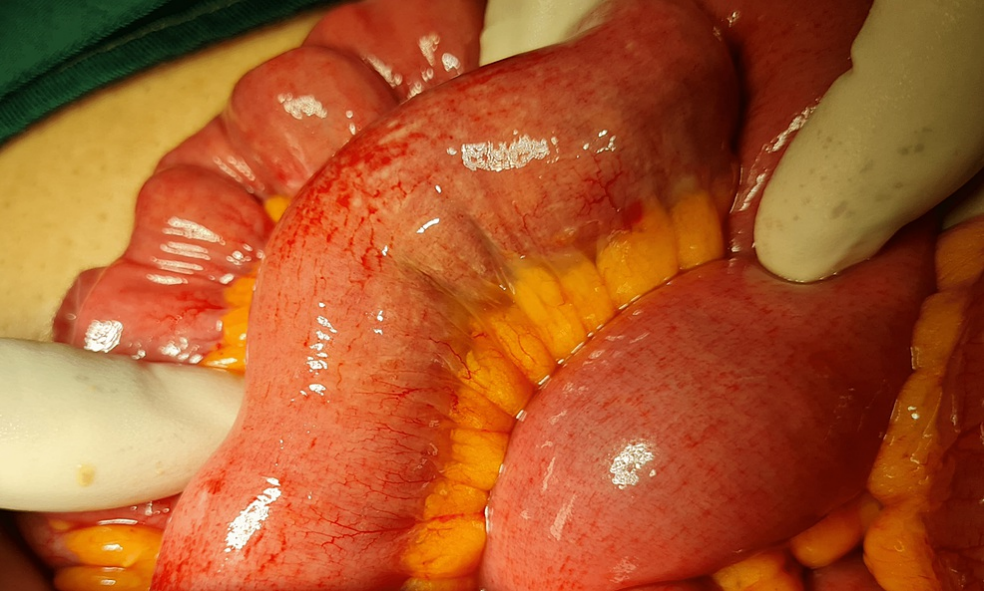
Edema of the gut

**Figure 3:**
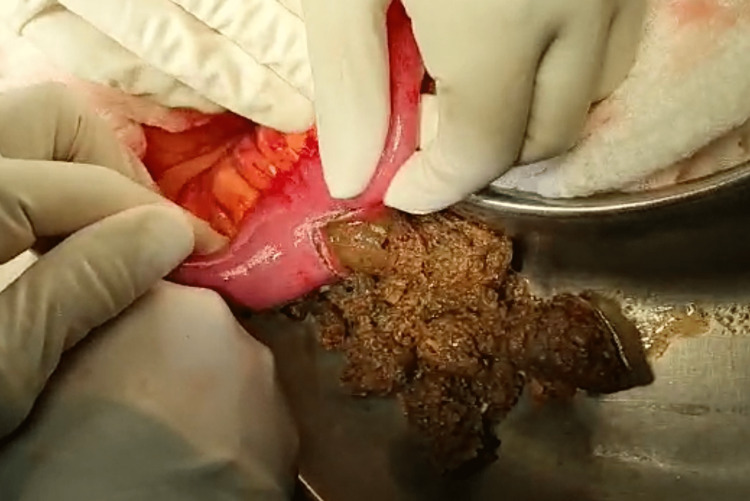
Ileotomy made in the healthy gut, with contents milked through

Biochemical screening for endocrine disorders revealed no abnormality. CT scan showed small bowel distention with a transition point in the distal ileum (Figure 1). His constipation and distension persisted, and a laparotomy was therefore planned. At surgery, the distal 40 cm of ileum was collapsed and 50 cm of ileum proximal to this was distended with a firm, tubular mass that was deformable on digital compression. The ileum itself was healthy, but in the distal 30 cm of the mass, there was edema of the gut (Figure 2). The ileum was incised nearer the proximal end of the mass, as the gut at that location was healthy (Figure 3).

**Figure 4:**
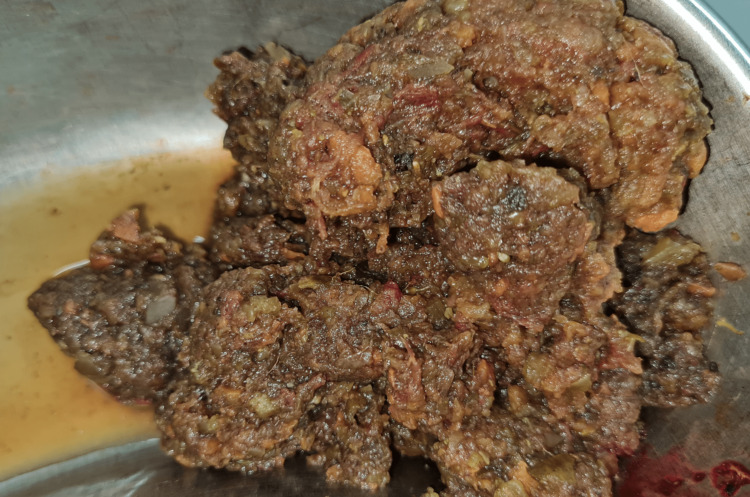
Thick, fibrous contents milked from the enterotomy

**Figure 5:**
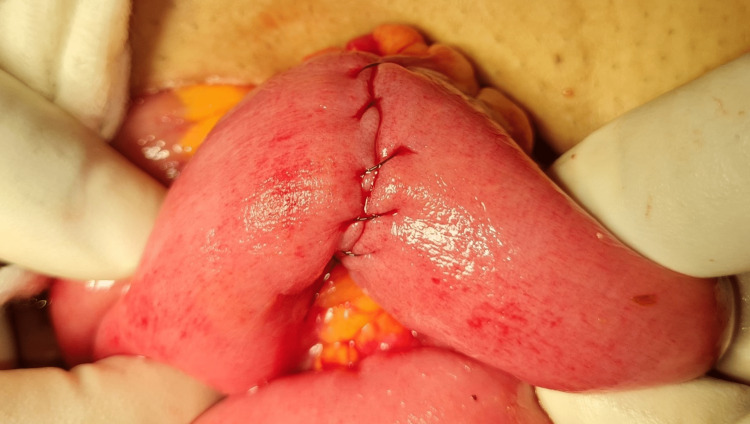
Transverse closure of the ileotomy

The obstructing contents were milked back, and firm, thick, sticky, fibrous material was delivered through the ileotomy (Figure 4). After emptying the gut, a 22 Fr Foley catheter was passed proximally and distally, washing out the contents to avoid postoperative impaction and recurrent obstruction. The ileotomy was closed transversely in two layers (Figure 5).

He recovered uneventfully and was discharged on the third postoperative day.

## Discussion

Small bowel obstruction secondary to bezoars is a relatively rare but well-documented phenomenon. In a review by Ghosheh et al., 0.8% of small bowel obstructions (SBOs) were caused by bezoars while Kement et al. reported that 7% of SBOs were caused by phytobezoars and Bedioui et al. reported 4% [1-3].

Obstruction, however, is not the only documented complication of small bowel bezoars. Mooghal et al. documented a case of impending ileal perforation secondary to bezoar with petechiae at the terminal ileum and a hard intraluminal mass at the same point [4]. Erzurumlu et al. reported two cases out of 34 who had a perforation of the ileum and subsequent peritonitis while Oh et al. documented one case of bowel necrosis [5,6].

All types of bezoars are classed by their composition. The commonest are phytobezoars, conglomerations of undigested plant matter. Celery, pumpkin, grape skins, prunes, raisins, and persimmons have been implicated in their formation, with the theory being that cellulose and tannins allow polymerization [7].

Kement et al. found that 36 out of 42 patients with intestinal obstruction due to bezoars had predisposing factors for bezoar formation [1]. One patient had a trichobezoar secondary to trichotillomania and all other patients had phytobezoars [1]. Previous gastric surgery was the number one cause of phytobezoar in 18 patients while large intakes of persimmon were documented in 17, mastication difficulties were noted in 16, and 12 patients had a history of diabetes mellitus [1]. Similarly, Yakan et al. found that 12 out of 14 patients had gastric surgery as their main predisposition while the other two had no teeth [8]. Erzurumlu et al. retrospectively found that prior gastric surgery was again the main risk factor (55.88%) [5]. Other factors were similar to Kement’s observations with persimmon intake accounting for 17.64% and diabetes mellitus for 11.76% [5]. However, our patient had none of these predisposing factors, had normal dentition, and practiced good mastication. His major change was dietary, involving much raw, plant-based, high-fiber food.

Diet changes have been implicated in the formation of bezoars, from increased intake of persimmons to swallowing pomegranate seeds and even ingestion of raw rhubarb [9-11]. The often-recommended high-fiber diets associated with good colon health have caused phytobezoars in patients with predisposing factors [12]. Escamilla et al. revealed in their retrospective study that most cases had previous gastric surgery and that 39.5% of the 87 cases reviewed had excessive fiber intake [13]. It is unusual for phytobezoars to occur without a predisposing factor; our patient's case of only a change in diet is rare.

Surgical options for intestinal bezoar management include milking the bezoar beyond the ileocecal valve, resection of the gut with primary anastomosis, and enterotomy with the removal of the obstruction and closure of the incision. Oh et al. reported that out of 20 patients, seven jejunal bezoars had jejunotomy and extraction of the bezoar [6]. In 11 ileal bezoars, eight had an ileotomy and extraction [6]. One case was resolved with milking of the bezoar fragments down the intestine and the last had an ileal resection and anastomosis when surgery revealed bowel necrosis [6]. Mooghal et al. also reported bowel resection and anastomosis secondary to impending perforation [4]. Erzurumlu et al. also had four cases of intestinal bezoars that required resection of the gut and anastomosis [5].

Our patient had the most common bezoar type, but his cause was unusual. The extreme change in his diet in the pursuit of a healthier lifestyle led to undigested vegetable matter accumulating in his gastrointestinal tract. The large volume combined with the vegetables being uncooked formed his bezoar, which could not be milked past the ileocecal valve because it was so dense. The gut, however, was viable so an enterotomy was made, the bezoar squeezed out of the incision, and the gut closed.

With the rising rate of obesity and the health consequences that come with it, interest in ‘fad diets’ have become more popular among the general population with internet searches for weight loss increasing between 2004 and 2018 [14-16]. Although weight loss is usually rapid with these diets, they come with their own complications often being nutritionally imbalanced, unmaintainable, and, in our patient, the complication of life-threatening intestinal obstruction from phytobezoar [17,18].

## Conclusions

Intestinal obstruction from phytobezoar is well-documented, but its occurrence because of extreme dieting is uncommon. Our patient’s case is unique, as none of the usual predisposing factors, gastric surgery, diabetes mellitus, or mastication difficulties, were present. A high-fiber diet was his only identifiable cause. We add to the current literature by documenting a phytobezoar and the resultant small bowel obstruction as consequences of excessive ‘healthy eating.’ This paper serves to alert both clinicians and patients to this risk from a high-fiber diet.
